# Supplementary material for: Clusters of longitudinal risk profile trajectories are associated with cardiometabolic diseases: Results from the population-based KORA cohort
Source: PLoS One. 2024 Mar 28;19(3):e0300966. doi: 10.1371/journal.pone.0300966 (PMC10977748; doi:10.1371/journal.pone.0300966)
Supplement: S1 File — (DOCX) [file pone.0300966.s001.docx]

# Supporting Information to

**Clusters of longitudinal risk profile trajectories are associated with cardiometabolic diseases: results from the population-based KORA cohort**

Fiona Niedermayer^1,2,3^, Gunther Schauberger^3^, Wolfgang Rathmann^4,5^, Stefanie J. Klug^3^, Barbara Thorand^2,4^, Annette Peters^1,2,4,6^, Susanne Rospleszcz^1,2,6,7^*

^1^ Chair of Epidemiology, IBE, Faculty of Medicine, LMU Munich, Munich, Germany

^2^ Institute of Epidemiology, Helmholtz Zentrum München, German Research Center for Environmental Health, Neuherberg, Germany

^3^ Chair of Epidemiology, Department of Sport and Health Sciences, Technical University of Munich, Munich, Germany

^4^ German Center for Diabetes Research (DZD), München-Neuherberg, Neuherberg, Germany

^5^ Department for Biometrics and Epidemiology, German Diabetes Research Institute, Leibniz Institute for Diabetes Research at Heinrich Heine University, Düsseldorf, Germany

^6^ German Center for Cardiovascular Disease Research (DZHK), Munich Heart Alliance, Munich, Germany

^7^ Department of Diagnostic and Interventional Radiology, University Medical Center Freiburg, Faculty of Medicine, University of Freiburg, Freiburg, Germany

*Corresponding author:

Email: [Susanne.rospleszcz@helmholtz-muenchen.de](mailto:Susanne.rospleszcz@helmholtz-muenchen.de) (SR)

**Additional tables**

**S1** **Table.** **Description of laboratory measurements at Exam 1, Exam 2 and Exam 3.**

| **Biomarker** |  | **Exam 1**  **(1999 - 2001)** | **Exam 2**  **(2006 - 2008)** | **Exam 3**  **(2013 - 2014)** |
| --- | --- | --- | --- | --- |
| **Total cholesterol** | Instrument | Hitachi 717 | Dimension RxL | Dimension Vista 1500 or^a^  Cobas c701/702 |
|  | Method & assay | Enzymatic, photometric CHOL | Enzymatic, colorimetric CHOL Flex | Enzymatic, colorimetric CHOL Flex or^a^  enzymatic, colorimetric CHOL2 |
|  | Manufacturer | Roche Diagnostics GmbH, Mannheim, Germany | Dade Behring, Newark USA | Siemens Healthcare Diagnostics  Inc., Newark, USA or^a^  Roche Diagnostics GmbH, Mannheim, Germany |
| **LDL cholesterol** | Instrument | Hitachi 717 | Dimension RxL | Dimension Vista 1500 or^a^  Cobas c701/702 |
|  | Method & assay | enzymatic, photometric CHOL | Enzymatic, colorimetric ALDL Flex | Enzymatic, colorimetric LDLC flex or^a^  enzymatic, colorimetric LDL_C |
|  | Manufacturer | Roche Diagnostics GmbH, Mannheim, Germany | Dade Behring, Newark USA | Siemens Healthcare Diagnostics Inc., Newark, USA or^a^  Roche Diagnostics GmbH, Mannheim, Germany |
| **HDL cholesterol** | Instrument | Hitachi 717 | Dimension RxL | Dimension Vista 1500 or^a^  Cobas c701/702 |
|  | Method & assay | Enzymatic, photometric CHOL | Enzymatic, colorimetric AHDL Flex | Enzymatic, colorimetric HDLC Flex or^a^  enzymatic, colorimetric HDLC3 |
|  | Manufacturer | Roche Diagnostics GmbH, Mannheim, Germany | Dade Behring Inc., Newark, USA | Siemens Healthcare Diagnostics Inc., Newark, USA or^a^  Roche Diagnostics GmbH, Mannheim, Germany |
| **HbA1c** | Instrument | Hitachi 717 | Adams HA 8160 Hemoglobin Analysis System | VARIANT II TURBO Hemoglobin Testing System |
|  | Method & assay | Turbidimetric inhibition immunoassay | Cation-exchange high performance liquid chromatographic, photometric assay | Cation-exchange high performance liquid  chromatographic, photometric VARIANT II TURBO HbA1c Kit - 2.0 |
|  | Manufacturer | Roche Diagnostics GmbH, Mannheim, Germany | Arkray Inc., distributed by A. Menarini Diagnostics, Florence, Italy | Bio-Rad Laboratories Inc., Hercules, USA |
| **Blood pressure** | Instrument | Digital (Type: HEM-705CP) | Digital (Type: HEM-705CP) | Digital (Type: HEM-705CP) |
|  | Manufacturer | OMRON HEALTHCARE GmbH | OMRON HEALTHCARE GmbH | OMRON HEALTHCARE GmbH |
| **Height Weight** | Instrument | scale (SECA 709) with integrated measuring rod (SECA 221) | measuring rod (SECA 240 or 242 (digital)) and scale (SECA 709) | scale (SECA 635 or 877) and  measuring rod (SECA 242) |
|  | Manufacturer | Seca GmbH & Co, KG, Hamburg | Seca GmbH & Co, KG, Hamburg | Seca GmbH & Co, KG, Hamburg |

^a^the KORA FF4 measurement instrument and assays changed from Siemens to Roche at about half of the study period. Based on 122 KORA FF4 samples, which were measured with both instruments / assays during the time of the method change, calibration formulas were developed. The Siemens measurement results were calibrated to correspond to the Roche measurements using the following formulas [all units in mg/dl]: Total_Cholesterol_Roche = 3.00 + Total_Cholesterol_Siemens * 1.00; HDL_Cholesterol_Roche = 2.40 + HDL_Cholesterol_Siemens * 1.12; LDL_Cholesterol_Roche = antilog (-0.13328 + log LDL_Cholesterol_Siemens * 1.03051); Triglycerides_Roche = 4.97073 + Triglycerides_Siemens * 0.90732

**S2 Table. The Calinski-Harabasz criterion for varying number of clusters determined by k-means clustering.**

| **Number of clusters** | **Male sample**  (n = 976) | **Female sample**  (n = 1004) |
| --- | --- | --- |
|  | Calinski-Harabasz criterion | Calinski-Harabasz criterion |
| **3** | **169.3** | **195.2** |
| 4 | 135.5 | 167.5 |
| 5 | 116.8 | 144.7 |
| 6 | 108.0 | 133.3 |
| 7 | 96.5 | 121.6 |
| 8 | 90.1 | 113.0 |

For k-means, the number of clusters has to be specified in advance. We therefore varied the cluster number from three to eight and chose the optimal number afterwards. To this aim, we used the Calinski-Harabasz criterion which basically considers the “within variance” and the “between variance” of clusters [29]. The cluster number with the highest Calinski-Harabasz criterion was chosen as the optimal cluster solution, since it indicates compact and well-separated clusters. According to the Calinski-Harabasz criterion after 20 rerolling steps, the optimal number of clusters was three.

**S3** **Table. Comparison of characteristics of excluded and included participants at Exam 1.**

|  | **Men** | | | | | **Women** | | | | |
| --- | --- | --- | --- | --- | --- | --- | --- | --- | --- | --- |
|  | **Excluded participants** | | **Included participants** | |  | **Excluded participants** | | **Included participants** | |  |
|  | N | Mean ± SD | N | Mean ± SD | p | N | Mean ± SD | N | Mean ± SD | p |
| Age [years] | 1110 | 51.4 ± 15.0 | 976 | 47.5 ± 12.6 | <0.001^b^ | 1161 | 50.4 ± 15.1 | 1004 | 46.9 ± 12.0 | <0.001^b^ |
| *Blood pressure (BP)* |  |  |  |  |  |  |  |  |  |  |
| Systolic BP [mmHg] | 1102 | 135.4 ± 19.2 | 976 | 132.6 ± 16.4 | <0.001^b^ | 1151 | 125.1 ± 20.2 | 1004 | 120.6 ± 17.7 | <0.001^b^ |
| Diastolic BP [mmHg] | 1102 | 82.7 ± 10.6 | 976 | 83.2 ± 10.1 | 0.284 | 1151 | 78.0 ± 10.1 | 1004 | 77.5 ± 9.9 | 0.335 |
| *Anthropometric factors* |  |  |  |  |  |  |  |  |  |  |
| Body-Mass Index [kg/m²] | 1096 | 27.7 ± 4.3 | 976 | 27.3 ± 3.8 | 0.013^b^ | 1138 | 27.4 ± 5.6 | 1004 | 26.4 ± 4.9 | <0.001^b^ |
| Weight [kg] | 1096 | 83.8 ± 3.6 | 976 | 84.3 ± 12.3 | 0.429^b^ | 1139 | 71.2 ± 14.1 | 1004 | 69.6 ± 13.0 | 0.007^b^ |
| Waist circumference [cm] | 1096 | 98.3 ± 11.5 | 976 | 96.4 ± 10.2 | <0.001^b^ | 1140 | 86.9 ± 13.4 | 1004 | 83.4 ± 11.7 | <0.001^b^ |
| *Lipids* |  |  |  |  |  |  |  |  |  |  |
| Total cholesterol [mg/dl] | 1089 | 229.7 ± 45.9 | 976 | 226.3 ± 41.4 | 0.079^b^ | 1142 | 229.6 ± 45.9 | 1004 | 223.2 ± 40.6 | 0.001^b^ |
| HDL cholesterol [mg/dl] | 1085 | 50.8 ± 13.9 | 976 | 51.3 ± 13.5 | 0.335 | 1140 | 63.1 ± 17.4 | 1004 | 65.3 ± 17.4 | 0.003 |
| LDL cholesterol [mg/dl] | 1082 | 143.6 ± 41.4 | 976 | 139.7 ± 39.4 | 0.030 | 1138 | 136.8 ± 43.9 | 1004 | 129.5 ± 39.7 | <0.001^b^ |
| Triglycerides [mg/dl] | 537 | 165.2 ± 122.9  Median: 131.0 | 360 | 157.4 ± 105.7  Median: 129.5 | 0.322^a^ | 525 | 141.8 ± 95.8  Median: 118.0 | 327 | 120.4 ± 65.3  Median: 106.0 | <0.001^a^ |
| *Glucose metabolism* |  |  |  |  |  |  |  |  |  |  |
| HbA1c [%] | 1088 | 5.6 ± 0.7 | 976 | 5.5 ± 0.6 | 0.008^b^ | 1138 | 5.6 ± 0.7 | 1004 | 5.6 ± 0.5 | 0.056^b^ |
| 2-hour glucose [mg/dl] | 407 | 133.3 ± 54.7  Median: 121.0 | 291 | 120.0 ± 49.4  Median: 108.0 | <0.001^a^ | 394 | 126.9 ± 48.0  Median: 119.0 | 260 | 119.1 ± 44.5  Median: 109.0 | 0.004^a^ |
| Fasting blood glucose [mg/dl] | 425 | 106.5 ± 19.3 | 293 | 103.6 ± 16.1 | 0.027^b^ | 413 | 100.2 ± 16.4 | 268 | 98.4 ± 14.3 | 0.144^b^ |
| *Inflammation* |  |  |  |  |  |  |  |  |  |  |
| C-reactive protein [mg/L] | 1064 | 2.8 ± 5.5  Median: 1.3 | 976 | 2.0 ± 3.6  Median: 0.9 | <0.001^a^ | 1111 | 2.9 ± 4.2  Median: 1.5 | 1004 | 2.7 ± 5.0  Median: 1.2 | 0.001^a^ |
| *Medication* |  |  |  |  |  |  |  |  |  |  |
| Antihypertensive | 1101 | 242 (22.0%) | 976 | 126 (12.9%) | <0.001 | 1158 | 276 (23.8%) | 1004 | 42 (4.2%) | <0.001 |
| Lipid-lowering | 1101 | 85 (7.7%) | 976 | 57 (5.8%) | 0.108 | 1158 | 67 (5.8%) | 1004 | 126 (12.6%) | 0.110 |
| *CVD risk* |  |  |  |  |  |  |  |  |  |  |
| Framingham risk score [%] | 987 | 19.6 ± 17.6 | 953 | 13.7 ± 12.4 | <0.001^b^ | 1093 | 9.1 ± 10.5 | 999 | 5.6 ± 6.0 | <0.001^b^ |
| *Lifestyle factors* |  |  |  |  |  |  |  |  |  |  |
| Alcohol consumption [g/day] | 1099 | 23.5 ± 26.9  Median: 17.1 | 976 | 23.7 ± 24.7  Median: 17.1 | 0.156^a^ | 1154 | 8.4 ± 13.9  Median: 2.9 | 1004 | 8.8 ± 12.0  Median: 2.9 | <0.001^a^ |
| Pack years^b^ | 1069 | 20.7 ± 25.4  Median: 13.0 | 976 | 14.7 ± 21.7  Median: 5.9 | <0.001^a^ | 1115 | 7.4 ± 13.3  Median: 0.0 | 1004 | 6.0 ± 10.2  Median: 0.0 | 0.748^a^ |
| Physically active | 1098 | 478 (43.5%) | 976 | 518 (53.1%) | <0.001 | 1154 | 521 (45.1%) | 1004 | 541 (53.9%) | <0.001 |
| Smoking | 1107 |  | 976 |  | <0.001 | 1159 |  | 1004 |  | 0.248 |
| never-smoker |  | 279 (25.2%) |  | 333 (34.1%) |  |  | 603 (52.0%) |  | 526 (52.4%) |  |
| ex-smoker |  | 429 (38.8%) |  | 399 (40.9%) |  |  | 290 (25.0%) |  | 274 (27.3%) |  |
| smoker |  | 399 (36.0%) |  | 244 (25.0%) |  |  | 266 (23.0%) |  | 204 (20.3%) |  |
| *Prevalent diseases* |  |  |  |  |  |  |  |  |  |  |
| T2D | 1110 | 82 (7.5%) | 976 | 29 (3.0%) | <0.001 | 1161 | 65 (5.6%) | 1004 | 17 (1.7%) | <0.001 |
| Myocardial infarction | 1110 | 49 (4.4%) | 976 | 20 (2.0%) | 0.004 | 1161 | 7 (0.6%) | 1004 | 2 (0.2%) | 0.262 |
| Stroke | 1110 | 32 (2.9%) | 976 | 5 (0.5%) | <0.001 | 1161 | 14 (1.2%) | 1004 | 3 (0.3%) | 0.032 |

Continuous variables are presented as mean ± standard deviation, p-values derived from two sample t-test indicate whether mean values differ significantly between included and excluded participants at Exam 1. Categorical variables are presented as counts and percentages, p-values are calculated by X^2^ test and indicate whether counts differ significantly between included and excluded participants at Exam 1.

^a^ Wilcoxon-Test was used due to skewed distribution. ^b^ Welch-Test was used due to non-equal variances.

**S4 Table.** **Number of prevalent cardiometabolic cases at each examination stratified by sex and cluster membership.**

|  | **Men (n = 976)** | | | **Women (n = 1004)** | | |
| --- | --- | --- | --- | --- | --- | --- |
|  | Cluster I  n = 310 | Cluster II  n = 346 | Cluster III  n = 320 | Cluster I  n = 371 | Cluster II  n = 231 | Cluster III  n = 402 |
| *Cardiometabolic cases* |  |  |  |  |  |  |
| Exam 1 (1999 - 2001) | 8 | 7 | 35 | **/** | / | 22 |
| Exam 2 (1999 - 2001) | 13 | 19 | 74 | 2 | 2 | 53 |
| Exam 3 (2013 -2014) | 16 | 30 | 124 | 8 | 7 | 97 |
| Follow-up (2016) | 19 | 41 | 138 | 10 | 13 | 100 |
| *Type 2 diabetes* |  |  |  |  |  |  |
| Exam 1 (1999 - 2001) | 3 | 4 | 22 | / | / | 17 |
| Exam 2 (1999 - 2001) | 5 | 11 | 55 | 1 | 1 | 46 |
| Exam 3 (2013 -2014) | 8 | 17 | 97 | 3 | 3 | 79 |
| Follow-up (2016) | 10 | 23 | 109 | 4 | 7 | 86 |
| *CVD cases* |  |  |  |  |  |  |
| Exam 1 (1999 - 2001) | 5 | 3 | 15 | / | / | 5 |
| Exam 2 (1999 - 2001) | 8 | 9 | 32 | 1 | 1 | 11 |
| Exam 3 (2013 -2014) | 8 | 14 | 53 | 5 | 4 | 25 |
| Follow-up (2016) | 10 | 23 | 61 | 6 | 6 | 30 |
|  |  |  |  |  |  |  |
| *Myocardial infarction* |  |  |  |  |  |  |
| Exam 1 (1999 - 2001) | 5 | 2 | 13 | / | / | 2 |
| Exam 2 (1999 - 2001) | 6 | 2 | 23 | / | / | 6 |
| Exam 3 (2013 -2014) | 6 | 6 | 35 | 1 | 1 | 9 |
| Follow-up (2016) | 7 | 14 | 39 | 1 | 2 | 13 |
| *Stroke* |  |  |  |  |  |  |
| Exam 1 (1999 - 2001) | 1 | 1 | 3 | / | / | 3 |
| Exam 2 (1999 - 2001) | 3 | 7 | 10 | 1 | 1 | 5 |
| Exam 3 (2013 -2014) | 3 | 9 | 22 | 4 | 3 | 16 |
| Follow-up (2016) | 4 | 11 | 26 | 5 | 4 | 24 |
| *CVD mortality* |  |  |  |  |  |  |
| Exam 1 (1999 - 2001) | / | / | / | / | / | / |
| Exam 2 (1999 - 2001) | / | / | / | / | / | / |
| Exam 3 (2013 -2014) | / | / | / | / | / | / |
| Follow-up (2016) | 1 | 2 | 7 | 1 | / | 2 |

**S5 Table.** **Men: Mean cardiometabolic risk profile for each cluster at Exam 1, Exam 2 and Exam 3 obtained by k-means.**

|  | **Exam** | **Cluster I**  **(n=310)** | **Cluster II (n=346)** | **Cluster III**  **(n = 320)** |
| --- | --- | --- | --- | --- |
| **Age [years]** | Exam 1 | 39.7 ± 11.3 | 48.2 ± 11.3 | 54.3 ± 10.7 |
|  | Exam 2 | 46.9 ± 11.3 | 55.4 ± 11.3 | 61.4 ± 10.6 |
|  | Exam 3 | 53.3 ± 11.3 | 61.8 ± 11.3 | 67.9 ± 10.6 |
| *Blood pressure (BP)* |  |  |  |  |
| **Systolic BP [mmHg]** | Exam 1 | 126.1 ± 13.0 | 131.4 ± 13.9 | 138.6 ± 15.5 |
|  | Exam 2 | 119.2 ± 13.0 | 127.6 ± 14.4 | 133.0 ± 15.0 |
|  | Exam 3 | 118.4 ± 12.8 | 125.6 ± 14.6 | 126.1 ± 15.5 |
| **Diastolic BP [mmHg]** | Exam 1 | 78.9 ± 8.5 | 83.1 ± 8.8 | 86.4 ± 9.4 |
|  | Exam 2 | 74.1 ± 8.3 | 79.1 ± 8.2 | 80.1 ± 9.2 |
|  | Exam 3 | 74.0 ± 7.9 | 76.4 ± 9.1 | 73.5 ± 10.0 |
| *Anthropometric factors* |  |  |  |  |
| **Body-Mass Index [kg/m²]** | Exam 1 | 24.5 ± 2.2 | 26.6 ± 2.2 | 30.2 ± 2.6 |
|  | Exam 2 | 25.0 ± 2.4 | 26.9 ± 2.3 | 31.1 ± 2.9 |
|  | Exam 3 | 25.4 ± 2.5 | 27.3 ± 2.7 | 31.3 ± 3.2 |
| Weight [kg] | Exam 1 | 77.2 ± 8.7 | 82.2 ± 9.0 | 82.2 ± 9.0 |
|  | Exam 2 | 79.4 ± 9.6 | 83.7 ± 9.8 | 83.7 ± 9.8 |
|  | Exam 3 | 80.4 ± 10.5 | 84.5 ± 11.0 | 84.5 ± 11.0 |
| **Waist circumference [cm]** | Exam 1 | 88.7 ± 6.6 | 94.8 ± 6.2 | 104.6 ± 6.7 |
|  | Exam 2 | 90.2 ± 7.4 | 97.0 ± 6.5 | 108.8 ± 7.5 |
|  | Exam 3 | 94.0 ± 7.9 | 100.8 ± 7.4 | 111.9 ± 8.3 |
| *Lipids* |  |  |  |  |
| **Total cholesterol [mg/dl]** | Exam 1 | 193.1 ± 27.5 | 252.8 ± 28.2 | 226.6 ± 35.5 |
|  | Exam 2 | 191.5 ± 25.5 | 238.6 ± 27.6 | 203.6 ± 31.7 |
|  | Exam 3 | 200.3 ± 29.8 | 235.6 ± 29.4 | 189.9 ± 33.5 |
| **HDL cholesterol [mg/dl]** | Exam 1 | 54.6 ± 12.4 | 51.7 ± 11.8 | 46.5 ± 11.9 |
|  | Exam 2 | 53.0 ± 11.7 | 50.6 ± 9.8 | 45.9 ± 9.8 |
|  | Exam 3 | 62.5 ± 15.1 | 58.3 ± 13.4 | 52.6 ± 13.6 |
| **LDL cholesterol [mg/dl]** | Exam 1 | 108.1 ± 25.7 | 164.9 ± 26.7 | 140.3 ± 34.2 |
|  | Exam 2 | 119.0 ± 23.2 | 159.3 ± 23.9 | 129.0 ± 27.3 |
|  | Exam 3 | 122.7 ± 26.2 | 157.6 ± 26.1 | 116.8 ± 30.0 |
| Triglycerides [mg/dl]^a^ | Exam 1 | 106.5 ± 80.3 | 147.6 ± 83.2 | 167.1 ± 84.5 |
|  | Exam 2 | 99.0 ± 54.4 | 142.7 ± 66.6 | 155.3 ± 67.0 |
|  | Exam 3 | 108.1 ± 55.2 | 138.1 ± 62.0 | 145.1 ± 65.8 |
| *Glucose metabolism* |  |  |  |  |
| **HbA1c [%]** | Exam 1 | 5.4 ± 0.3 | 5.4 ± 0.3 | 5.6 ± 0.3 |
|  | Exam 2 | 5.3 ± 0.3 | 5.4 ± 0.3 | 5.7 ± 0.4 |
|  | Exam 3 | 5.3 ± 0.4 | 5.5 ± 0.4 | 5.8 ± 0.5 |
| 2-hour glucose [mg/dl]^a^ | Exam 1 | 110.4 ± 37.2 | 105.4 ± 32.5 | 126.8 ± 40.0 |
|  | Exam 2 | 95.3 ± 24.4 | 107.5 ± 27.3 | 130.4 ± 31.8 |
|  | Exam 3 | 99.0 ± 27.4 | 113.8 ± 34.0 | 136.1 ± 35.0 |
| Fasting blood glucose [mg/dl]^a^ | Exam 1 | 98.0 ± 9.1 | 99.6 ± 9.5 | 105.2 ± 10.7 |
|  | Exam 2 | 94.0 ± 8.5 | 97.6 ± 10.1 | 106.3 ± 13.6 |
|  | Exam 3 | 98.2 ± 9.8 | 102.3 ± 12.0 | 113.2 ± 17.3 |
| *Inflammation* |  |  |  |  |
| C-reactive protein [mg/L] | Exam 1 | 1.1 ± 1.5 | 1.7 ± 1.9 | 2.2 ± 2.1 |
|  | Exam 2 | 1.1 ± 1.2 | 1.7 ± 1.6 | 2.0 ± 1.7 |
|  | Exam 3 | 1.2 ± 1.5 | 1.9 ± 1.9 | 2.4 ± 2.1 |
| *Medication* |  |  |  |  |
| Antihypertensive | Exam 1 | 17 (5.5%) | 23 (6.6%) | 86 (26.9%) |
|  | Exam 2 | 36 (11.6%) | 63 (18.2%) | 172 (53.8%) |
|  | Exam 3 | 48 (15.5%) | 103 (29.8%) | 213 (66.6%) |
| Lipid-lowering | Exam 1 | 15 (4.8%) | 7 (2.0%) | 35 (10.9%) |
|  | Exam 2 | 21 (6.8%) | 26 (7.5%) | 74 (23.1%) |
|  | Exam 3 | 18 (5.8%) | 51 (14.7%) | 119 (37.2%) |
| *CVD risk* |  |  |  |  |
| Framingham risk score [%] | Exam 1 | 5.8 ± 6.5 | 13.8 ± 10.3 | 21.6 ± 14.1 |
|  | Exam 2 | 7.8 ± 7.6 | 18.3 ± 12.4 | 26.0 ± 16.2 |
|  | Exam 3 | 10.0 ± 8.4 | 21.3 ± 13.1 | 27.5 ± 15.3 |
| *Lifestyle factors* |  |  |  |  |
| Alcohol consumption [g/day] | Exam 1 | 19.1 ± 19.1 | 24.6 ± 20.6 | 23.2 ± 22.0 |
|  | Exam 2 | 18.5 ± 19.1 | 23.0 ± 20.3 | 20.1 ± 21.3 |
|  | Exam 3 | 19.8 ± 18.6 | 23.2 ± 20.8 | 17.8 ± 19.7 |
| Pack years^b^ | Exam 1 | 8.8 ± 12.7 | 13.3 ± 16.7 | 18.3 ± 20.1 |
|  | Exam 2 | 10.2 ± 14.1 | 14.9 ± 18.5 | 19.9 ± 22.0 |
|  | Exam 3 | 11.1 ± 15.4 | 15.7 ± 19.4 | 20.4 ± 22.5 |
| Physically active | Exam 1 | 198 (63.9%) | 197 (56.9%) | 123 (38.4%) |
|  | Exam 2 | 194 (62.6%) | 207 (59.8%) | 157 (49.1%) |
|  | Exam 3 | 200 (64.5%) | 206 (59.5%) | 144 (45.0%) |
| Smoking |  |  |  |  |
| never-smoker | Exam 1 | 125 (40.3%) | 108 (31.2%) | 100 (31.3%) |
|  | Exam 2 | 125 (40.3%) | 108 (31.2%) | 100 (31.3%) |
|  | Exam 3 | 125 (40.3%) | 108 (31.2%) | 100 (31.3%) |
| ex-smoker | Exam 1 | 91 (29.4%) | 152 (43.9%) | 156 (48.8%) |
|  | Exam 2 | 112 (36.1%) | 164 (47.4%) | 183 (57.2%) |
|  | Exam 3 | 119 (38.4%) | 177 (51.2%) | 190 (59.4%) |
| smoker | Exam 1 | 94 (30.3%) | 86 (24.9%) | 64 (20.0%) |
|  | Exam 2 | 73 (23.5%) | 74 (21.4%) | 37 (11.6%) |
|  | Exam 3 | 66 (21.3%) | 61 (17.6%) | 30 (9.4%) |

Continuous variables are presented as mean ± standard deviation, categorical variables are presented as counts and percentages. Variables written in bold were used for clustering.

^a^Sample size deviate

**S6 Table.** **Women: Mean cardiometabolic risk profile for each cluster at Exam 1, Exam 2 and Exam 3 obtained by k-means.**

|  | **Exam** | **Cluster I (n=371)** | **Cluster II**  **(n=231)** | **Cluster III**  **(n = 402)** |
| --- | --- | --- | --- | --- |
| **Age [years]** | Exam 1 | 41.4 ± 10.4 | 39.3 ± 8.0 | 56.5 ± 8.5 |
|  | Exam 2 | 48.5 ± 10.4 | 46.4 ± 7.9 | 63.5 ± 8.4 |
|  | Exam 3 | 55.0 ± 10.4 | 52.9 ± 7.9 | 70.0 ± 8.4 |
| *Blood pressure (BP)* |  |  |  |  |
| **Systolic BP [mmHg]** | Exam 1 | 112.2 ± 13.3 | 115.7 ± 13.3 | 129.7 ± 15.4 |
|  | Exam 2 | 107.4 ± 13.0 | 112.4 ± 13.1 | 123.7 ± 13.7 |
|  | Exam 3 | 106.5 ± 13.5 | 113.0 ± 13.6 | 120.4 ± 15.3 |
| **Diastolic BP [mmHg]** | Exam 1 | 73.1 ± 8.4 | 77.1 ± 8.3 | 81.1 ± 8.7 |
|  | Exam 2 | 69.0 ± 7.8 | 73.6 ± 7.7 | 75.4 ± 8.5 |
|  | Exam 3 | 68.3 ± 7.4 | 73.6 ± 7.4 | 70.6 ± 8.6 |
| *Anthropometric factors* |  |  |  |  |
| **Body-Mass Index [kg/m²]** | Exam 1 | 22.5 ± 2.2 | 27.1 ± 3.6 | 29.2 ± 4.0 |
|  | Exam 2 | 22.7 ± 2.2 | 27.7 ± 3.6 | 29.8 ± 4.1 |
|  | Exam 3 | 23.1 ± 2.4 | 29.0 ± 4.0 | 30.1 ± 4.3 |
| Weight [kg] | Exam 1 | 60.2 ± 6.7 | 72.9 ± 10.4 | 75.2 ± 10.9 |
|  | Exam 2 | 61.1 ± 6.9 | 75.1 ± 10.5 | 76.8 ± 11.4 |
|  | Exam 3 | 61.7 ± 7.7 | 77.8 ± 11.3 | 76.6 ± 12.0 |
| **Waist circumference [cm]** | Exam 1 | 73.5 ± 5.5 | 84.6 ± 8.3 | 90.9 ± 9.1 |
|  | Exam 2 | 75.6 ± 6.0 | 88.7 ± 8.7 | 95.3 ± 10.1 |
|  | Exam 3 | 79.4 ± 7.0 | 95.2 ± 9.9 | 99.2 ± 10.2 |
| *Lipids* |  |  |  |  |
| **Total cholesterol [mg/dl]** | Exam 1 | 209.5 ± 33.3 | 201.3 ± 32.1 | 245.8 ± 32.7 |
|  | Exam 2 | 205.6 ± 34.3 | 199.5 ± 28.3 | 236.7 ± 33.5 |
|  | Exam 3 | 216.8 ± 34.8 | 211.3 ± 32.1 | 233.6 ± 37.3 |
| **HDL cholesterol [mg/dl]** | Exam 1 | 73.3 ± 14.4 | 56.5 ± 12.9 | 61.8 ± 16.0 |
|  | Exam 2 | 69.0 ± 11.8 | 54.4 ± 10.1 | 59.4 ± 13.1 |
|  | Exam 3 | 83.2 ± 14.8 | 62.1 ± 12.7 | 69.2 ± 17.3 |
| **LDL cholesterol [mg/dl]** | Exam 1 | 108.9 ± 32.8 | 116.6 ± 30.4 | 153.2 ± 30.1 |
|  | Exam 2 | 117.3 ± 30.0 | 125.2 ± 25.6 | 151.5 ± 29.7 |
|  | Exam 3 | 123.1 ± 30.3 | 133.1 ± 30.2 | 147.6 ± 33.8 |
| Triglycerides [mg/dl]^a^ | Exam 1 | 89.2 ± 41.1 | 100.9 ± 66.0 | 124.0 ± 50.2 |
|  | Exam 2 | 71.3 ± 29.1 | 95.2 ± 41.4 | 124.3 ± 47.2 |
|  | Exam 3 | 83.2 ± 33.2 | 109.4 ± 45.4 | 125.4 ± 44.5 |
| *Glucose metabolism* |  |  |  |  |
| **HbA1c [%]** | Exam 1 | 5.5 ± 0.3 | 5.4 ± 0.3 | 5.7 ± 0.3 |
|  | Exam 2 | 5.3 ± 0.3 | 5.3 ± 0.3 | 5.7 ± 0.4 |
|  | Exam 3 | 5.3 ± 0.3 | 5.3 ± 0.3 | 5.8 ± 0.4 |
| 2-hour glucose [mg/dl]^a^ | Exam 1 | 97.5 ± 26.9 | 113.0 ± 35.9 | 121.1 ± 36.0 |
|  | Exam 2 | 92.0 ± 23.6 | 98.7 ± 23.7 | 123.5 ± 31.6 |
|  | Exam 3 | 92.4 ± 22.3 | 103.2 ± 25.8 | 127.8 ± 32.1 |
| Fasting blood glucose [mg/dl]^a^ | Exam 1 | 91.6 ± 7.6 | 90.2 ± 4.8 | 98.9 ± 10.1 |
|  | Exam 2 | 87.2 ± 7.4 | 89.1 ± 7.2 | 98.4 ± 10.4 |
|  | Exam 3 | 90.9 ± 8.6 | 94.4 ± 9.2 | 105.3 ± 13.8 |
| *Inflammation* |  |  |  |  |
| C-reactive protein [mg/L] | Exam 1 | 1.5 ± 2.0 | 2.3 ± 2.5 | 3.0 ± 2.7 |
|  | Exam 2 | 1.1 ± 1.4 | 2.1 ± 2.1 | 2.6 ± 2.1 |
|  | Exam 3 | 1.3 ± 1.7 | 2.3 ± 2.2 | 2.9 ± 2.5 |
| *Medication* |  |  |  |  |
| Antihypertensive | Exam 1 | 7 (1.9%) | 14 (6.1%) | 105 (26.1%) |
|  | Exam 2 | 26 (7.0%) | 30 (13.0%) | 176 (43.8%) |
|  | Exam 3 | 50 (13.5%) | 54 (23.4%) | 246 (61.2%) |
| Lipid-lowering | Exam 1 | 5 (1.3%) | 2 (0.9%) | 35 (8.7%) |
|  | Exam 2 | 15 (4.0%) | 11 (4.8%) | 69 (17.2%) |
|  | Exam 3 | 22 (5.9%) | 16 (6.9%) | 104 (25.9%) |
| *CVD risk* |  |  |  |  |
| Framingham risk score [%] | Exam 1 | 2.6 ± 2.5 | 2.7 ± 2.2 | 10.2 ± 7.0 |
|  | Exam 2 | 3.1 ± 2.8 | 3.7 ± 3.1 | 11.9 ± 7.7 |
|  | Exam 3 | 3.7 ± 3.0 | 5.1 ± 3.9 | 13.6 ± 8.7 |
| *Lifestyle factors* |  |  |  |  |
| Alcohol consumption [g/day] | Exam 1 | 9.5 ± 10.5 | 7.1 ± 9.6 | 7.7 ± 10.0 |
|  | Exam 2 | 8.8 ± 10.3 | 6.1 ± 8.9 | 7.0 ± 9.9 |
|  | Exam 3 | 9.6 ± 10.7 | 6.1 ± 9.5 | 7.2 ± 10.3 |
| Pack years^b^ | Exam 1 | 5.7 ± 8.2 | 6.5 ± 8.5 | 4.5 ± 8.1 |
|  | Exam 2 | 6.7 ± 9.5 | 7.6 ± 10.1 | 4.9 ± 9.0 |
|  | Exam 3 | 7.3 ± 10.5 | 8.4 ± 11.3 | 5.2 ± 9.8 |
| Physically active | Exam 1 | 229 (61.7%) | 116 (50.2%) | 196 (48.8%) |
|  | Exam 2 | 240 (64.7%) | 128 (55.4%) | 229 (57.0%) |
|  | Exam 3 | 261 (70.4%) | 134 (58.0%) | 199 (49.5%) |
| Smoking |  |  |  |  |
| never-smoker | Exam 1 | 175 (47.2%) | 97 (42.0%) | 254 (63.2%) |
|  | Exam 2 | 173 (46.6%) | 97 (42.0%) | 254 (63.2%) |
|  | Exam 3 | 170 (45.8%) | 97 (42.0%) | 254 (63.2%) |
| ex-smoker | Exam 1 | 102 (27.5%) | 78 (33.8%) | 94 (23.4%) |
|  | Exam 2 | 132 (35.6%) | 83 (35.9%) | 113 (28.1%) |
|  | Exam 3 | 147 (39.6%) | 83 (35.9%) | 117 (29.1%) |
| smoker | Exam 1 | 94 (25.3%) | 56 (24.2%) | 54 (13.4%) |
|  | Exam 2 | 66 (17.8%) | 51 (22.1%) | 35 (8.7%) |
|  | Exam 3 | 54 (14.6%) | 51 (22.1%) | 31 (7.7%) |

Continuous variables are presented as mean ± standard deviation, categorical variables are presented as counts and percentages. Variables written in bold were used for clustering.

^a^Sample size deviate

**Additional figures**

**
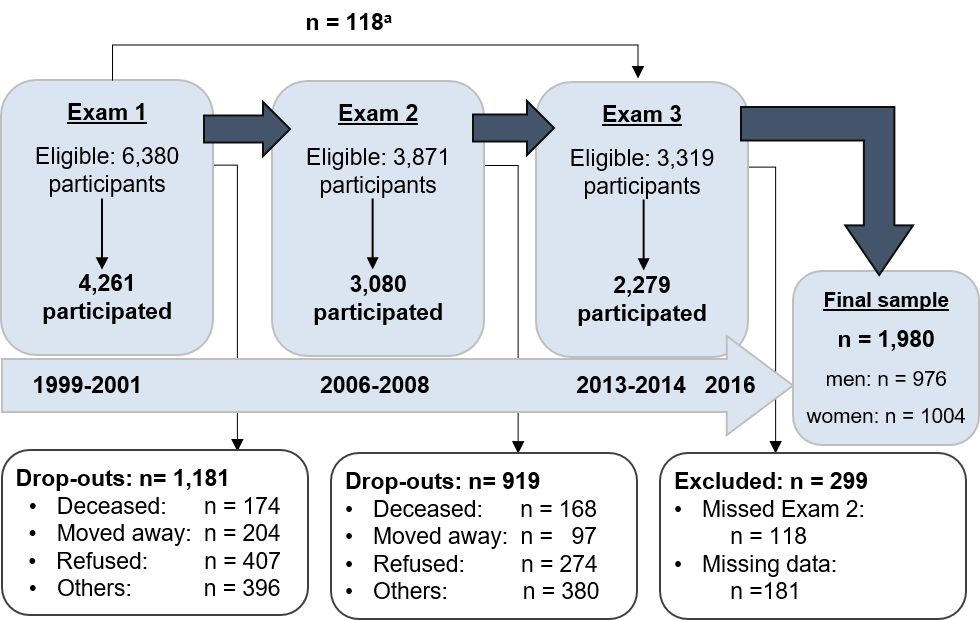
S1 Fig. Flowchart of the KORA cohort design and the main sample for the present analysis.** ^a^118 participants left out Exam 2;


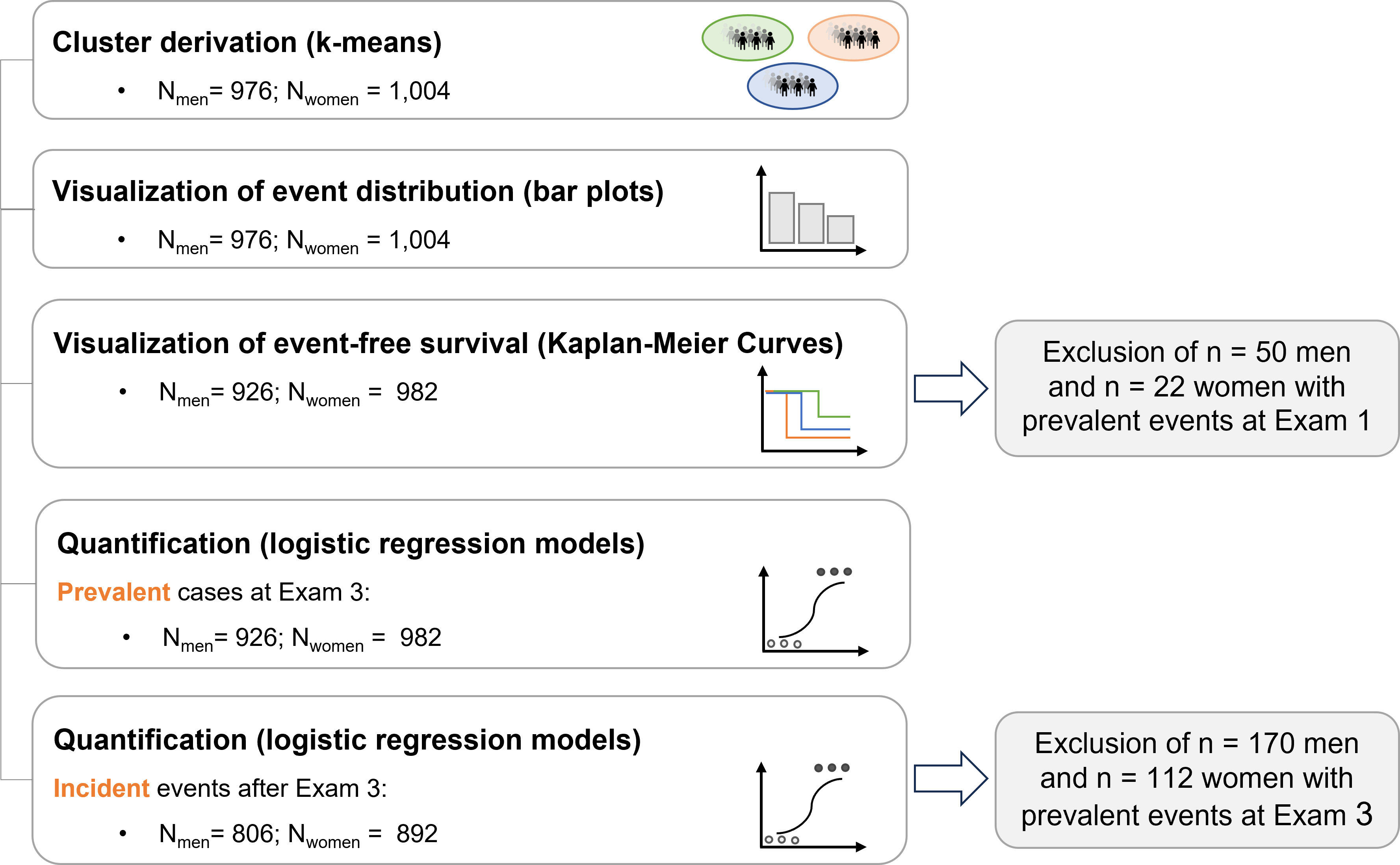


**S2 Fig. Diagram presenting sample size for each analytical step.**


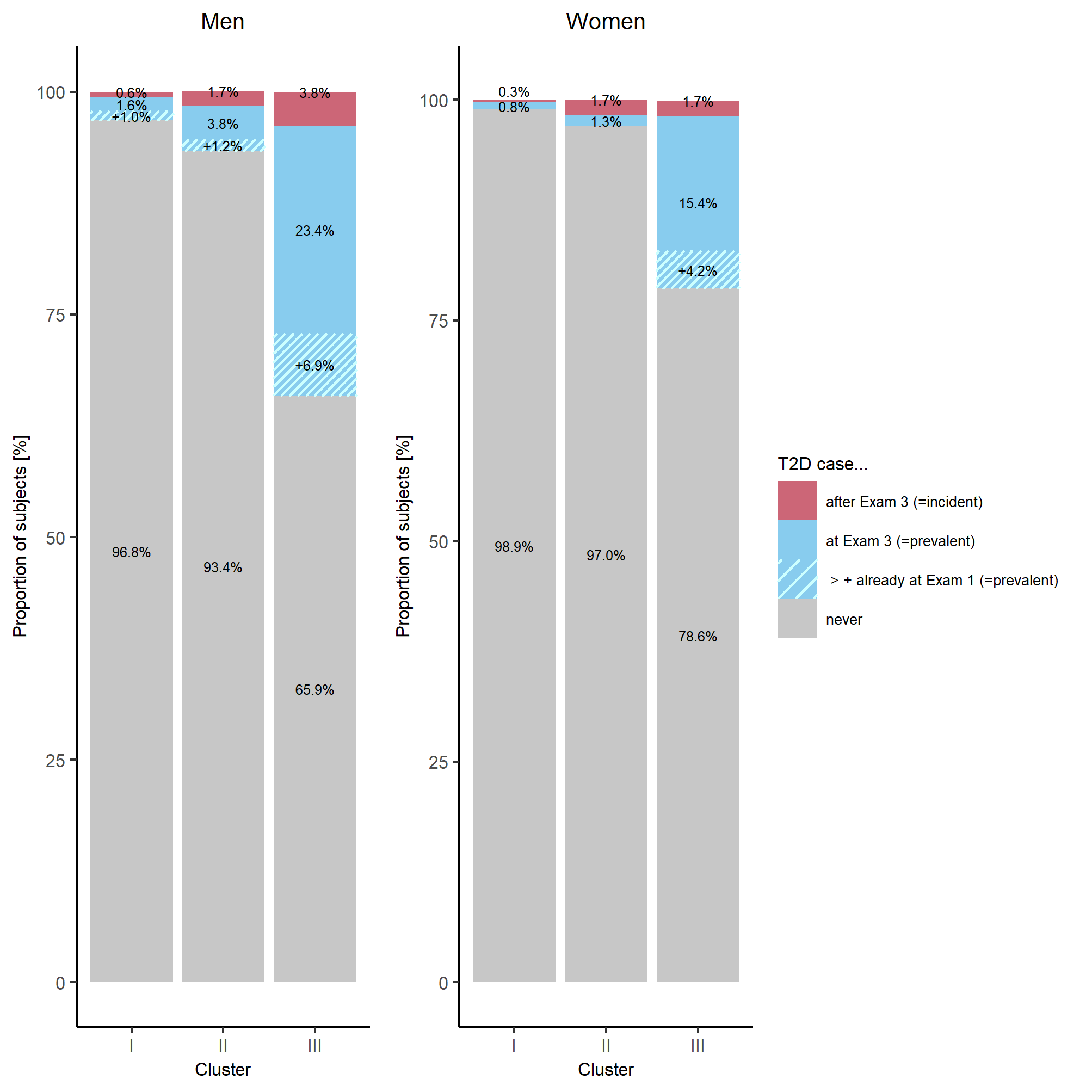
 **S3 Fig** **Distribution of prevalent and incident T2D cases according to cluster membership.**


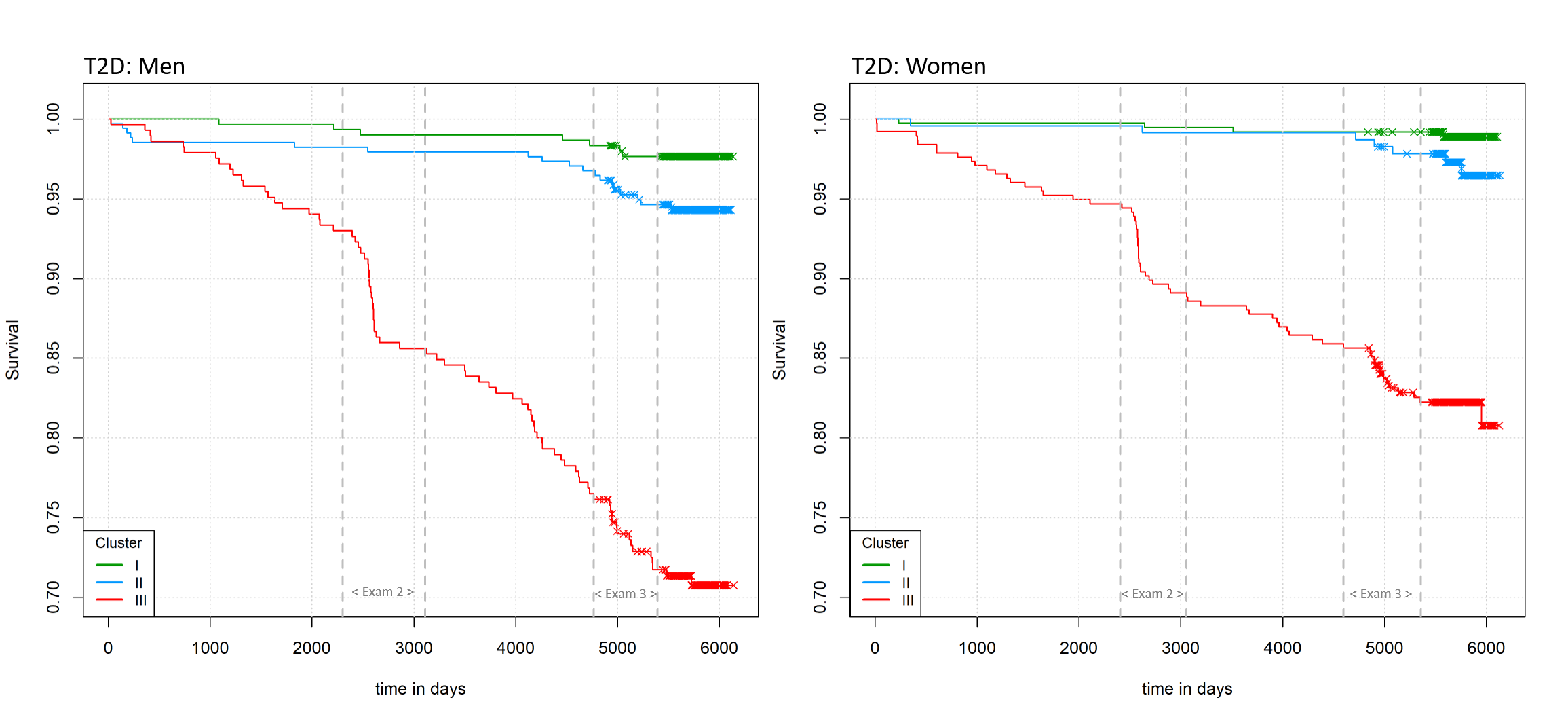


**S4 Fig. Kaplan-Meier curves showing T2D event-free survival stratified by cluster**. On the y-axis: probability of event-free survival without any cardiometabolic event, stratified by cluster membership. On the x-axis: Time from Exam1 until und of follow-up (3 years after Exam 3) in days. Periods of Exam 2 and 3 are marked by dashed grey lines. Marked decreases during the examination periods are mainly due to newly identified T2D cases that were ascertained during the OGTT performed as part of the examinations. For the visualization of Kaplan-Meier curves, prevalent cardiometabolic events at Exam 1 were excluded, resulting in a sample size of n = 926 for men and n = 982 for women.


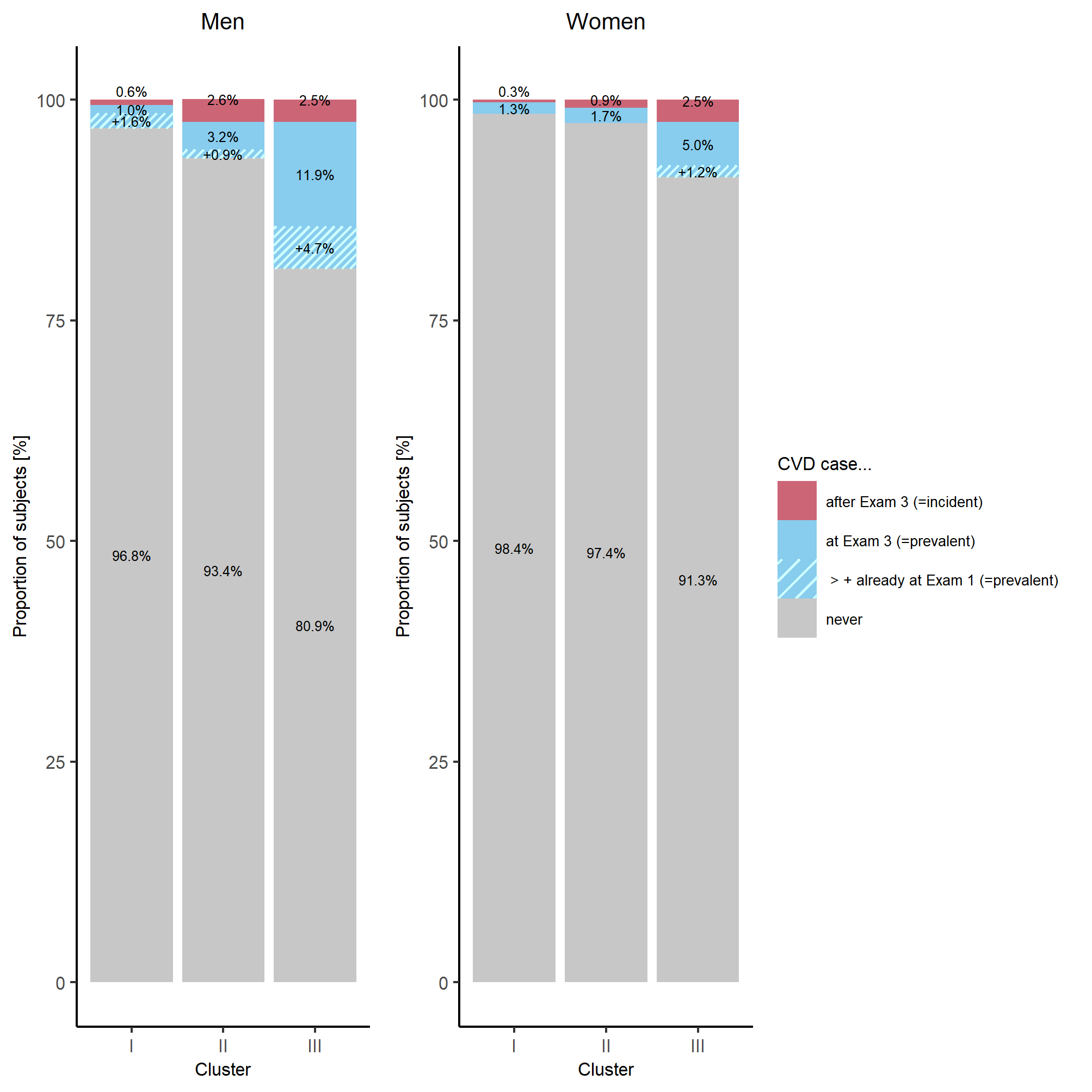
**S5 Fig. Distribution of prevalent and incident CVD cases according to cluster membership.**


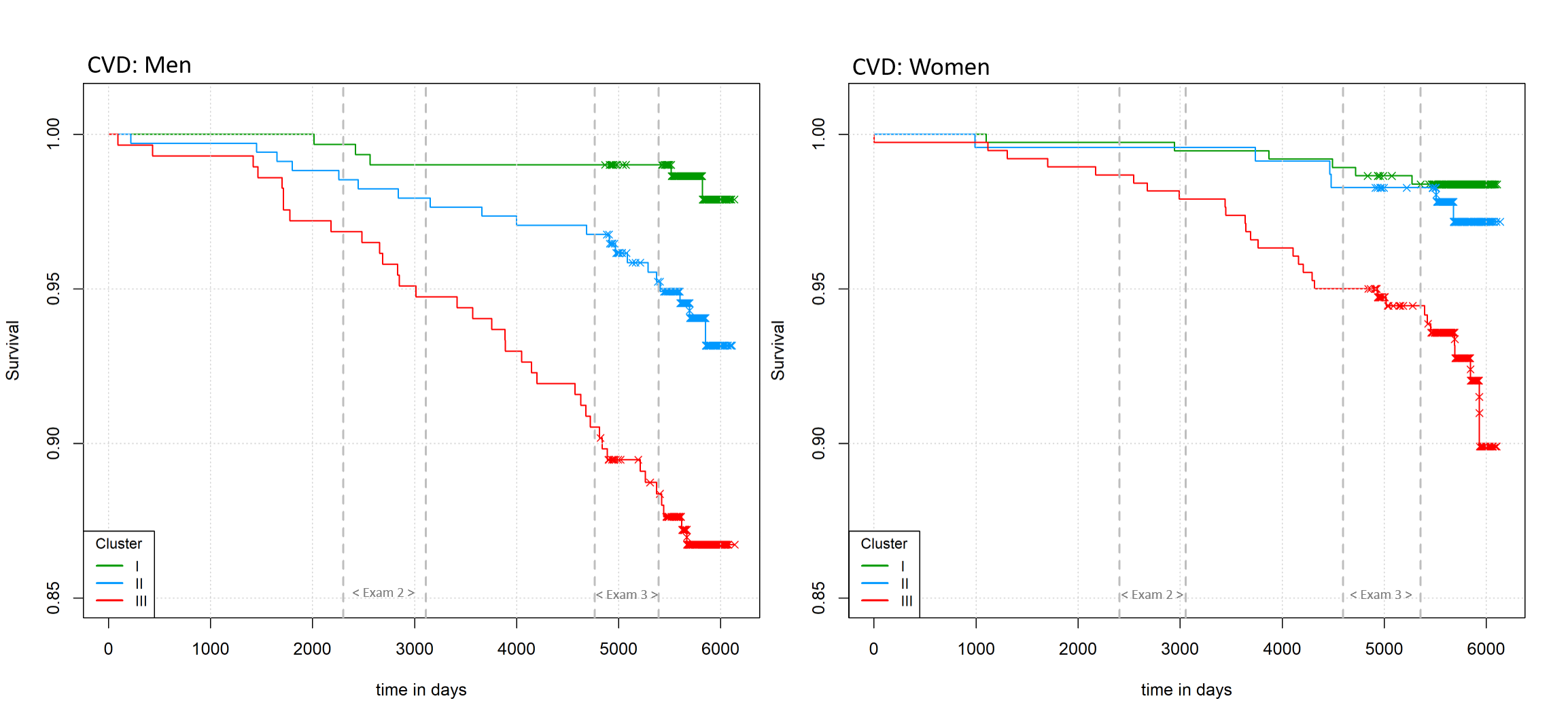


**S6 Fig.** **Kaplan-Meier curves showing CVD event-free survival stratified by cluster.** CVD events include fatal and non-fatal stroke, myocardial infarction and death from CVD. On the y-axis: probability of event-free survival without any cardiometabolic event, stratified by cluster membership. On the x-axis: Time from Exam1 until und of follow-up (3 years after Exam 3) in days. Periods of Exam 2 and 3 are marked by dashed grey lines. For the visualization of Kaplan-Meier curves, prevalent cardiometabolic events at Exam 1 were excluded, resulting in a sample size of n = 926 for men and n = 982 for women.
